# Drug Delivery Systems for Vitamin D Supplementation and Therapy

**DOI:** 10.3390/pharmaceutics11070347

**Published:** 2019-07-18

**Authors:** Eliza Glowka, Joanna Stasiak, Janina Lulek

**Affiliations:** Department of Pharmaceutical Technology, Poznan University of Medical Sciences, 6 Grunwaldzka Street, 60-780 Poznan, Poland

**Keywords:** vitamin D, cholecalciferol, drug delivery systems, targeted delivery, nanoparticles, food fortification, cancer

## Abstract

Vitamin D (VD) is a fat-soluble prohormone well known for its role in regulating calcium and phosphate metabolism. It has been clinically used for many years to prevent rickets in children, osteomalacia, and osteoporosis in adults. VD insufficiency is a common medical condition, and many supplements are available in the market in order to increase serum 25-hydroxy VD levels to recommended amounts. Over the course of the last decades, it has become increasingly clear that calcitriol, an active form of VD, regulates multiple cellular processes with effects on normal and malignant cell growth and differentiation, and on the immune and cardiovascular function. Increasing evidence supports the role of the VD system in cancer prevention and therapy. Due to many pleiotropic and beneficial effects in extra-skeletal disorders, VD has gained potential and become an interesting active for encapsulation into drug delivery systems. The purpose of this review is to present the diversity of drug delivery systems that have been reported for VD or VD derivatives in an orderly manner across the following categories: Oral administration, application on the skin, cancer prevention/therapy, and other diseases or routes of administration.

## 1. Introduction

**Figure 1:**
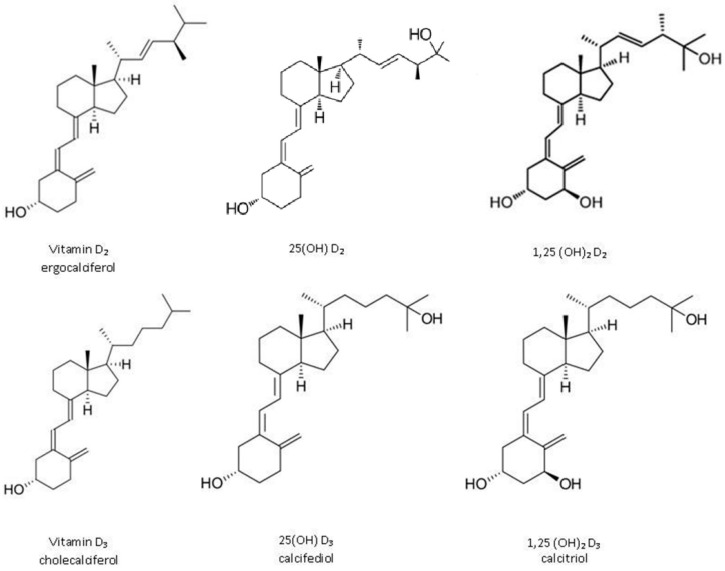
The chemical structure of ergocalciferol, cholecalciferol, and their active derivatives.

**Figure 2:**
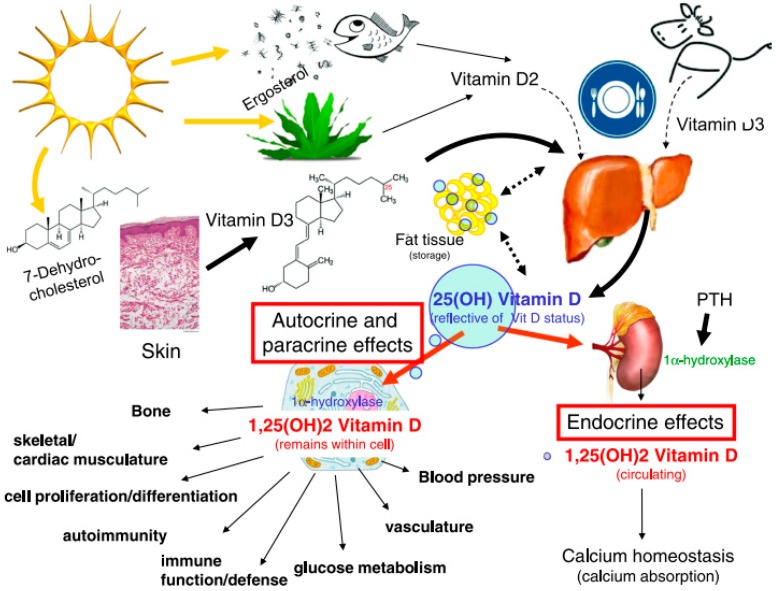
Scheme of vitamin D sources, fate in the body, and pleiotropic actions of 1,25-dihydroxy vitamin D (calcitriol), the active form of vitamin D. Reproduced with permission from [14]; published by Elsevier, 2011.

Vitamin D (VD) is one of the lipophilic vitamins. The most important forms of VD are cholecalciferol (vitamin D3, VD3) and ergocalciferol (vitamin D2, VD2) (Figure 1). VD3 is the main form and is available in some natural dietary products (egg yolk, flesh of fatty fish, and fish liver oils), food fortified with VD, and many forms of dietary supplements. VD2 is of plant origin and present in low amounts, e.g., in some mushrooms. VD2, being less potent than VD3, is rarely present in commercial preparations and fortified food. Despite that, it is a good alternative for vegans and vegetarians. However, the main source of VD is endogenous synthesis from 7-dehydrocholesterol in the human skin after sun exposure. Part of VD is stored in adipose and muscle tissue, and part of it gets hydroxylated. Independent of the source, VD3 and VD2 act as hormone precursors since they require two stages of metabolism: First to 25-hydroxy VD (25(OH)D, calcidiol) in the liver; then to 1α,25-dihydroxy VD (1,25(OH)D, calcitriol) in the kidney [1]. 25(OH)D3 is significantly less active than calcitriol and is transported in the circulation by binding to VD binding proteins. The amount of circulating 25(OH)D3 is the most reliable measurement and is thought to reflect body VD status best. The chemical structures of the vitamin and its active derivatives are presented in Figure 1, whereas the fate of VD in the body is presented in Figure 2. 

It is considered that most people are insufficient or deficient in VD due to a lack of sun exposure, extensive use of sunscreens, which block VD synthesis, and poor dietary intake. Maintaining recommended serum levels, i.e., 30–60 ng/mL of 25(OH)D3, can be achieved through vitamin supplementation or food fortification without changing lifestyle to avoid impaired skeletal and overall health [2].

Nowadays, a lot is known and written about the mechanism of action and the beneficial effects of VD on the human body [1,3,4]. Similar to other steroid hormones, the active form of VD binds to a nuclear receptor (VDR, vitamin D receptor) and modulates gene expression. As the VDR is present in most cells in the body and calcitriol directly or indirectly regulates as much as 3–5% of the human genome, VD activity is widespread, and it exerts actions that can limit the progression of multiple diseases [5]. One of the most important functions of VD is to maintain skeletal calcium and phosphate balance, and VD deficiency can result in lower mineral density. For this reason, VD has been used in order to prevent or treat rickets in children, osteomalacia, osteoporosis, bone fractures, and secondary hyperparathyroidism in adults. There is also evidence that VD can prevent or has beneficial effects in many other diseases such as malignancies, diabetes, and cardiovascular or autoimmune diseases [3]. A few drugs with VD-like activity (VD analogues), with more or less pronounced calcemic action (e.g., calcifediol, alfacalcidiol, paricalcitol, calcitriol, eldecalcitol), are also clinically available and used topically to treat psoriasis due to the regulation of keratinocytes proliferation, differentiation, and apoptosis [6]. 

Nowadays, the use of VD or VD analogues in prophylaxis or in the therapy of many beyond-skeletal diseases seems to be very interesting, especially for possible use in cancer treatment [7,8,9]. In such a case, the calcemic activity becomes undesirable and targeting delivery to cancer cells could be expected. However, anti-proliferative activity is associated with high concentrations of calcitriol at which hypercalcemia may be induced due to the action of calcitriol in stimulating intestinal calcium absorption. Additionally, the activity of the hormone is self-regulated as it simultaneously induces its inactivation [5]. To overcome these barriers, several analogues of calcitriol with lower calcemic activities that activate the target receptor have been synthesised [10,11,12]. The analogues should be selective and should have increased pro-differentiating and anti-proliferative properties. Therefore, it is necessary to characterize new VD analogues fully. Some of the analogues have undergone evaluation in preclinical or clinical trials for potential anticancer activity [11]. It is worth paying attention to seocalcitol (EB 1089), which is one of the potential analogues. Seocalcitol is 50–200 times more potent than calcitriol concerning regulation of cell growth and differentiation in vitro as well as in vivo. It has reduced calcemic activity in vivo compared to that of calcitriol, but a lower binding affinity for the VDR [13].

Many attempts have been made to harness some drug delivery systems (DDS) to deliver VD or VD analogues for skeletal or non-skeletal effects. A drug delivery system is defined as a formulation or a device that enables the introduction of a therapeutic substance into the body and improves its efficacy and safety by controlling the rate, time, and place of drug release. Ideal DDS should increase the bioavailability of the drug, provide for controlled or sustained drug delivery, and transport the drug intact to the site of action, while avoiding the non-diseased host tissues. The product should be safe and reliable, cost effective, stable, easy to administer to the patients, and drug delivery should be maintained under various physiological variables [15]. Meeting all these requirements at the same time is often impossible and not always necessary. The development of DDS has been going on for a few decades (starting from the discovery of liposomes in the 1960s) and this advanced drug delivery is promising much in the treatment of disease, with superior control of delivery and action over conventional formulations. However, development of such a delivery system with zero-order release kinetics, suitable stability, and ease of scaling up is a difficult task. Therefore, the vast majority of potential applications of DDS remain at the initial research stage.

**Figure 3:**
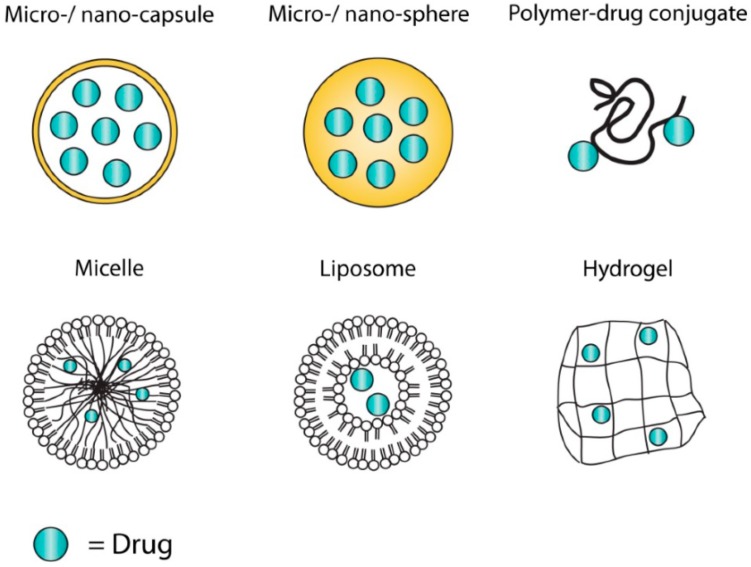
Different structures of drug delivery systems (not-exhaustive). Each structure has its advantages and disadvantages to incorporate and release different types of drugs. Reproduced with permission from [18]; published by MDPI, 2014.

Nowadays, the diversity of DDS is enormous, including nano- and microparticulate DDS that may be composed of a range of materials such as polymers, lipids, surfactants, and inorganic or hybrid materials [15,16,17]. The structure of the selected DDS is shown in Figure 3.

The recent increase in VD interest by the general public has fuelled a big rise in sales of over-the-counter VD preparations. Additionally, products with progressively increasing content of VD have been introduced with similar rapidity. Many types of pharmaceutical preparations for VD supplementation are commercially available, including oily drops, capsules, and tablets. At the same time, considering the potential use of VD and its analogues in the therapy of various diseases, they have become interesting candidates for encapsulation into DDS. This review of the published literature was conducted to present various types of delivery systems loaded with VD or VD-related compounds across the following categories: Oral administration (food fortification), application on the skin, cancer prevention/treatment, and finally other diseases or routes of administration.

## 2. Drug Delivery Systems for Oral Administration (Food Fortification)

Most dietary products are a poor source of VD, including breast milk. VD is minimally found in fat-free and low-fat dairy products, which are in growing demand. VD intake is found to be very low in vegetarians and vegans. The high phytate and fiber content of vegetarian diets may also reduce VD absorption [19]. Since VD insufficiency is a widespread public health problem, functional food fortified with this vitamin during processing has received increasing attention in recent years. While the use of oral VD supplements is a low-cost and practical method to treat deficiency, clinical advancements in VD oral administration are limited by its lipophilic character, low solubility in gastrointestinal fluid, and, thus, low bioavailability. Moreover, the facile degradation of VD by light, air, and heat should also be considered during manufacturing, storage, and use. In a moderate climate, VD supplementation should take place every day for most of the year. Thus, patient compliance could be low. Thereby, there is a need to develop new formulations of VD, especially those with prolonged release. In this context, the everyday consumption of foods enriched with VD seems to be a simpler and cheaper alternative.

In the gastrointestinal tract, after release from the food matrix or supplements, VD is included in micelles made by bile acids and enters the enterocytes via passive diffusion through an unsaturable mechanism [20]. Oral VD absorption requires three trans-membrane proteins that primarily function as cholesterol transporters in the intestine. Afterwards, it is included in chylomicrons and is then activated by the liver and kidneys. The absorption is blocked when there are not enough lipids in the intestine [19]. To improve VD bioavailability, it is important to enhance the water solubility [21]. Grossmann and Tangpricha (2010) reviewed a few studies that examined the effect of oil, cellulose, lactose, and ethanol vehicles on VD bioavailability from supplements. It was identified that VD in an oil vehicle has better oral bioavailability and produces a greater 25(OH)D response than VD in a powder (lactose or cellulose based) or an ethanol vehicle in healthy subjects [22]. However, a recent clinical trial concluded that oily drops (peanut oil) and capsules in lactose excipient, both containing VD, were bioequivalent [23].

### 2.1. Delivery Systems from a Food Processing Point of View

While the encapsulation of bioactive compounds in food products using nano/microparticulate systems is not a new concept [24], fortification of foods or beverages with VD still represents a big challenge for several reasons, including (i) high hydrophobicity that prevents direct dispersion of the vitamin in an aqueous food matrix, (ii) chemical degradation of VD leading to the reduction of functionality and bioavailability, and (iii) variable oral bioavailability [19,25,26]. In the vitamin structure (Figure 1), there are double bonds sensitive to oxidation. As mentioned earlier, light, oxygen, and high temperature induce vitamin isomerization and degradation into its inactive forms [22]. All these obstacles should be overcome to develop an efficient and inexpensive technology. Encapsulation into colloidal delivery systems could be a good solution, as it can either protect the vitamin against harmful conditions or make the vitamin soluble in aqueous systems. Various submicron-sized delivery systems are in particular interest. Over past years the popularity of nanotechnology in the food sector is increasing. 

On the basis of the literature search, it is evident that most delivery systems for VD have been developed for potential application in the food industry. In the field of food technology, VD is often considered as a model nutraceutical, as it has more than nutritional value on health. The term “nutraceutical” simply combines the words “nutrition” and “pharmaceutical”; however, up to today, there is no official definition distinct from, e.g., dietary supplements. The importance of nanotechnology in food processing can be evaluated by considering its role in the improvement of food product quality in terms of food texture, appearance, taste, nutritional value of the food, and food shelf-life [27]. Colloidal systems such as micelles, liposomes, nanoemulsions, and solid nanoparticles have found numerous applications as delivery vehicles for active pharmaceutical ingredients. Their use as vehicles for functional food ingredients is relatively new in the food industry, and it is especially promising to improve the bioavailability of poorly soluble substances such as functional lipids (e.g., carotenoids, phytosterols, polyunsaturated fatty acids, fat-soluble vitamins) and natural antioxidants [21]. 

One of the main aspects of nutraceutical encapsulation is the selection of safe material for encapsulation and procedure, preferably without using toxic solvents. Several new methods and materials that are safe to be used in the food industry have been developed from natural materials for nanoparticle production [28]. Delivery systems are commonly classified according to the main building blocks, which are necessarily GRAS (generally recognized as safe) food materials, preferably natural, and ideally-endogenous components of the product, thereby shortening the ingredient list and minimizing changes to the product sensory attributes, for higher consumer satisfaction. 

There are four main groups of delivery systems in food: Protein-based, carbohydrate-based, lipid-based, and mixed systems. Due to the negative health implications of high-fat consumption, there is a need for enriching low-fat and non-fat food and drinks with health-promoting oil-soluble bioactives, preferably using non-lipid, amphiphilic delivery systems [29]. It is obvious that delivery systems for such food application must be fabricated entirely from food-grade ingredients using economically viable processing operations and should provide suitable release of the nutraceuticals and their physical and chemical stability over the range of environmental conditions (e.g., temperature fluctuations, light, oxygen, and mechanical forces) that food and beverage products are usually exposed to during their manufacture, storage, and utilization. Additionally, the delivery system should not adversely influence the physicochemical or sensory properties of the food or beverage product that it is incorporated into, e.g., appearance, texture, or flavor [30].

According to the recommendations, the average daily requirement for VD for an adult is 1000–2000 international units (IU) [4]. This vitamin is a good candidate for encapsulation into drug delivery systems due to high activity (1 IU is equal to 0.025 µg), the relatively low daily requirement (25–50 µg/day), and high lipophilicity. Besides, most carriers are lipophilic in nature and generally the more lipophilic the active, the better encapsulation efficiency, and the lower the undesirable release. While the encapsulation of VD has been reported since 1993 [31], many disadvantages still exist, inhibiting further application and industrialization of VD supplementation or food fortification. For instance, encapsulation technology may involve the use of high temperature during the preparation, which would cause loss of VD activity, or the use of toxic solvents, which would pose potential side effects to final products due to residues of these solvents. Other disadvantages may include low loading capacity and incomplete encapsulation due to adsorption on the carrier surface resulting in little protection against UV exposure [32]. Lack of long-term stability of some delivery systems is also a serious technological problem. It should be mentioned that food and beverage products vary in their pH, ionic composition, ingredient interactions, storage conditions, and preparation procedures. Consequently, it will be important to test the stability of both the vitamin and the delivery system under the precise conditions under which they will be utilized in food products [26].

### 2.2. Types of Vitamin D Delivery Systems for Food Fortification 

Various types of delivery systems developed mainly over the last ten years for VD food fortification are described below or presented in Table 1. It was noticed that emulsions/nanoemulsions and nanoparticles are predominant types of vehicles for VD encapsulation. One of the most convenient means to incorporate lipophilic active components into aqueous media, so that they are suitable for utilization within food and beverage products, is to encapsulate them within colloidal lipid-based delivery systems. Such carriers may enhance VD bioaccessibility by forming mixed micelles in the small intestine that can solubilize and transport it [33]. 

There is increasing interest in the utilization of nanoemulsions in the food industry. Nanoemulsions are colloidal dispersions comprising two immiscible liquids with droplet sizes of the internal phase between 20 and 200 nm and are expected to improve oral bioavailability of poorly water-soluble drugs. Reducing droplet size in emulsion-based delivery systems has several consequences that may be beneficial for certain food applications: (i) Greater stability to droplet aggregation, and gravitational separation; (ii) higher optical clarity; and (iii) increased oral bioavailability. In particular, nanoemulsions may be particularly useful for encapsulating lipophilic nutraceuticals into aqueous-based food products that should be optically clear, such as fortified waters, soft drinks, or juices [26]. Additionally, nanoemulsions can be produced with natural food ingredients using simple production methods. Studies have shown that nanoemulsion composition effects lipid digestion and bioavailability so that the nature of the carrier oil is of great importance [34].

The most common technique used for the production of emulsion-type delivery systems is high-pressure homogenization. However, Guttoff et al. (2015) [26] proposed a spontaneous emulsification method that is simple and inexpensive to carry out, and therefore has great potential for forming nanoemulsion-based delivery systems for food, personal care, and pharmaceutical applications. Nevertheless, the method needs a higher surfactant concentration and may be less applicable to large-scale industrial processes [26].

An interesting approach has been taken by Salvia-Trujillo et al. (2017) [33]. They produced three VD2-enriched O/W emulsions with different particle size distributions (D4,3 values of 0.112, 0.53, and 14.5 μm) using different homogenization methods. To assess VD2 bioaccessibility (the fraction that is solubilized within the mixed micelle phase after lipid digestion), both an in-vitro gastrointestinal tract model consisting of mouth, stomach, and small intestine phases and in-vivo studies in rats were conducted. The in-vitro studies showed that smaller lipid droplets were digested more rapidly than larger ones and highest VD2 bioaccessibility was observed for the emulsions containing the smallest droplets. In contrast, the in-vivo rat feeding studies demonstrated that the absorption of VD2 was the highest for the emulsions containing the largest droplets [33].

Another of the most studied types of delivery systems for VD encapsulation is nanoparticles. Nanoencapsulation of nutraceuticals may be primarily used for better absorption, better stability, or sustained or even targeted delivery without affecting color or taste of food. Nanoparticles can be dispersed homogeneously and uniformly in the food system. They have more suitable properties compared to microstructures and can provide better bioavailability. There is a great number of synthetic polymers, biopolymers, and lipids available for the pharmaceutical, cosmetic, and food industries that may be used for the production of nanoparticles. However, only inexpensive food materials and technologies have the potential for commercialization. Abbasi et al. (2015) proposed whey protein isolate, which is a by-product of the dairy industry produced vastly in the world. Whey protein isolates have good nutritional and functional properties and can entrap hydrophobic compounds successfully. Furthermore, they can be digested and release their contents easily. Nanoparticles of whey protein isolate could entrap VD3 and delay its degradation in the presence of oxygen [28].

It is worth mentioning studies reported by researchers who prepared nanoparticles from hydrophobic alginate derivative (oleoyl alginate ester, OAE) loaded with VD3. They proved that such nanoparticles released in a sustained way in simulated gastric and intestinal fluid [21]. Next, they visualized the internalization of fluorescent VD3-loaded OAE nanoparticles by Caco-2 cells. Incorporation into OAE nanoparticles resulted in increased absorption of VD3 in mice. In in-vivo studies in rats with nutritionally induced VD-deficiency rickets, encapsulated VD3 had better efficacy than that of the VD3-free drug. Their studies provided evidence that OAE nanoparticles were valuable as nutraceutical delivery vehicles to enhance the absorption of VD3 [35].

An interesting approach was also presented by Semo et al. (2007). They proposed the use of bovine casein micelles for encapsulation and stabilization of VD2 that could enrich non-fat or low-fat food products. The authors demonstrated that these re-assembled casein micelles could provide protection against photochemical VD2 degradation [48]. In the next step, the authors improved the previous encapsulation technique by introducing ultra-high pressure homogenization and proved the protection conferred by the micelles to incorporated VD3 against heat-induced degradation and during cold storage. Finally, the bioavailability of a single high dose of 50,000 IU VD3 encapsulated in casein micelles in 1%-fat milk was at least as high as that using an aqueous Tween 80-emulsified VD3 supplement [25]. In another study, the authors compared the VD3 bioavailability in casein micelles to that in Tween 80, both in fat-free yogurt (VD3 dose: 50,000 IU), measuring serum 25(OH)D increase in subjects. No significant differences in mean 25(OH)D levels were observed, evidencing the fact that VD3 bioavailability in casein micelles was as high as that in the synthetic emulsifier [49]. Another recent clinical study found that the bioavailability of VD in casein micelles in the absence of fat was found to be insignificantly different from its bioavailability within fat, suggesting that the bioavailability of lipophilic bioactives in protein nanovehicles is not lower than in fat [50].

In the studies of Mohammadi et al. (2017), VD was incorporated into nanostructured lipid carriers (mean diameter of approximately 88 nm), and then the effect of rising 25(OH)D in rat blood was assessed. Nanoparticles demonstrated the faster and prolonged increase of 25(OH)D in plasma than the oily solution formulation of VD [43]. A different approach was proposed by Diarrassouba et al. (2015). They used food proteins (β-lactoglobulin and egg white lysozyme) to entrap VD3 and obtained microspheres (mean diameter of approximately 7 µm) by electrostatic interactions with an encapsulation efficiency of about 91%. The authors suggested the use of food proteins as potential food-grade vehicles for bioactives in the formulation of food products and pharmaceuticals [51].

Finally, as presented in Table 1, many other attempts were also carried out to improve water solubility, stability, and bioavailability of VD using different encapsulation strategies, like protein-based delivery systems [32,39,41], solid lipid [42] and nanostructured lipid carriers [43,44], liposomes [46], and microparticle [47]. Interestingly, most researchers underline the fact that VD is a vitamin sensitive to external factors. However, only some of the authors reported results concerning VD chemical stability studies. In such studies, the chemical stability of the vitamin is measured either by HPLC [37] or spectrophotometrically [19] and expressed as encapsulation efficiency decrease during storage of the formulation. Both report that the chemical stability of VD2 or VD3 loaded in oil-in-water nanoemulsions was dependent on the type of emulsifier; however, in these studies, there was no comparison to the stability of non-encapsulated VD, and the effect was not sufficient for long-term storage. 

## 3. Drug Delivery Systems for Application on the Skin 

Alternatively to the oral administration of VD, which can have low efficiency because of many barriers and differences of conditions along the route, skin application may provide several advantages. Transdermal delivery gives a chance to avoid the first-pass effect of the liver, ensures the release for a long period, and could be for people with diseases that impair the oral absorption of VD3. On the other side, the topical application could provide a high concentration of the active substance in the upper skin layers that is essential for the treatment of several skin diseases typified by the disfunction of keratinocytes in the lower epidermis, mainly psoriasis [52]. Analyzing the research described in the literature in which VD/VD analogue was loaded in drug delivery systems, most potential applications were designed for topical administration on the skin. However, a few interesting studies on transdermal VD delivery were also conducted.

### 3.1. Transdermal Delivery

The transdermal route of permeation of vitamin D3 may be interesting for people with fat malabsorption in the intestine, due to Crohn’s disease, gastric bypass, bile acid-binding medications, cystic fibrosis, and celiac disease. However, the transdermal route has little use, and there is little scientific literature on the subject. D’Angelo Costa and co-workers [53] investigated the feasibleness of VD3 skin retention and permeation with the presence of chemical penetration enhancers (soybean lecithin, isopropyl palmitate, propylene glycol, ethoxydiglycol, and cereal alcohol) at different pharmaceutical forms (gel and cream) through human skin from abdominal surgery. However, according to their results, gel formulation showed VD3 detection at stratum corneum in 4 h and epidermis and dermis in 24 h, whereas VD3 from the cream was detected only at the skin surface (no VD3 was found at receptor fluid for both formulations). They concluded that VD3 did not indicate feasibleness for transdermal use since it did not permeate through human skin, probably due to high lipophilicity and, thus, high affinity with the vehicle in the cream. Nevertheless, the transdermal route could be more effective with less lipophilic derivates of VD3 and with a different combination of penetration enhancers in the gel [53]. In the research of Alsaqr et al. [54], ointments with oleic acid and dodecylamine as penetration enhancers were used to evaluate the transdermal delivery of VD3 through porcine skin in 24 h. The results were retention at stratum corneum and epidermis for three formulations assessed (control, oleic acid, and dodecylamine), but VD was detected in the receptor medium only in the case of formulation with dodecylamine. They concluded that the transdermal route could be effective. However, it should be noted that porcine skin has a higher permeability due to the greater amount of hair follicles than human skin [54].

The above results obtained for transdermal VD delivery with conventional formulations (ointments and gels) suggest that drug delivery systems could be attractive to enhance such delivery. To our knowledge, the report of Bubshait et al. (2018) is the only research on humans in this area that so far has been published [55]. They did a prospective randomized controlled trial in which patients in the study group were given Top-D (VD3 gel made from proniosomal technology, 1 g contained 5000 IU of VD3) to apply daily on the skin, while the control group was given 1 g of *Aloe vera* gel to be applied every day. All the patients had VD deficiency (25OHD level ≤ 20 ng/mL). After four months, only in the study group, the serum 25OHD level was sufficient (25OHD level 37.17 ± 6.04 ng/mL). However, the proniosomal technology was not described in detail, and the only information is that the average particle size of VD carriers is about 3.8 µm. Hence, the absorption of vitamin D3 through the pores occurred without difficulty, as skin pores are approximately 50 µm in diameter [55]. Already in 2014, this group of researchers from the same center did a randomized controlled pilot study on the transdermal route of vitamin D3 and received similar results. One group of patients applied a topical formulation with *Aloe vera* and VD3, and another group applied formulation without VD3. After three months, an increase in the 25OHD level was observed [56]. In this study, aromatic oils and glycerin were used as penetration enhancers, and the results were comparable to those obtained for proniosomal technology.

Untypical studies were conducted by Kim et al. (2018) in which, first, VD was encapsulated into poly(lactic-*co*-glycolic acid) (PLGA) nanoparticles by a well-known emulsion solvent evaporation method; then, nanoparticles were immobilized onto the microneedles by a dip-coating method. The overall system was designed for transdermal VD delivery as an alternation for oral supplementation. It should be explained that microneedles easily pierce the skin layer with enough mechanical strength and allow the localization of drugs within the dermal region. Finally, layers of nanoparticles coated on solid microneedles were dissolved entirely into the intradermal region in the porcine skin model and revealed better performance for VD3 release into the receptor compartment compared to the ointment-based transdermal method [57].

Finally, an interesting study was done by Yamagishi et al., who evaluated the effects of a reservoir-type calcitriol transdermal patch on plasma calcitriol and calcium concentrations in dairy cattle. Interestingly, VD3, or its active form (calcitriol), is used as a prophylaxis for parturient hypocalcemia in dairy cows. The patches were applied to the skin of the tail for two days at intervals of at least three weeks. The data of this study describe evidence of measurable transdermal absorption of exogenous calcitriol from a patch and its sufficient biological action to elevate plasma calcium concentrations [58].

### 3.2. Topical Delivery

VD and its analogues have been proven to have anti-psoriatic activity by decreasing the proliferation of epidermal keratinocytes, by regulating the differentiation process and apoptosis of keratinocytes, by regulating the cutaneous immune system, and through their anti-inflammatory ability [6,59]. The effectiveness on psoriasis of VD and its derivatives (calcitriol, calcipotriol, calcipotriene, tacalcitol, hexafluoro-1,25(OH)D, and maxacalcitol) has been known since 1985, being confirmed in numerous trials. The therapy with VD is one of the most popularly prescribed topical medications for this disease as the first-line, singly or in combination with topical corticosteroids, and numerous studies have documented the efficacy of such treatment [6].

Such activity in the skin is due to the presence of VD3 receptors on keratinocytes and fibroblasts and an autonomous VD3 pathway in which VD3 is converted to calcitriol, the hormonally active form. Nevertheless, the VD3 derivatives show also some hypercalcemic activity, which can be a limitation. That is why a significant challenge is to increase the limited drug penetration and to minimize systemic exposure [60,61]. Another issue that should be taken into consideration when developing topical delivery systems is the high sensitivity of VD3 to external environmental factors such as light, heat, and oxygen [57]. The success of drug delivery depends, among other things, on the physicochemical properties of the active and its carriers in the formulation. Several studies have been focused on using nanoparticles for topical delivery of VD or VD analogues, including liposomes, solid lipid nanoparticles, polymeric nanospheres, or combinations of PLGA nanoparticles and microneedles [52]. Table 2 summarizes the delivery systems and methods of preparation described below.

The idea of liposomal encapsulation for topical administration of VD derivatives is not new. For keeping small the negative side effects of VD3 derivatives and for increasing the drug concentration in the skin, Merz et al. (1994) investigated their incorporation in liposomes to optimize their use for psoriasis treatment; however, no in-vitro or ex-vivo study was done. They prepared liposomes made of synthetic or naturally occurring lipids and evaluated the incorporation of VD and VD analogues into liposomes and their thermal properties. Their results showed that VD3 and its analogues incorporated into the lipid bilayer, altering the lipid–lipid interactions in the liposomes. The incorporation rates were generally found to be high, i.e., ≥80% [62]. It is crucial to consider that liposomes which have a phase transition temperature (T_m_) above the temperature of the skin (32 °C) will be in a rigid gel state and these with a T_m_ below 32 °C will be in a fluid liquid state. Several studies have presented that liposomes in a liquid state have improved penetration into the skin. It was also proven that the liposome-mediated delivery of calcipotriol to the epidermis of diseased skin is also affected by the fluidity of the liposomal membrane [63].

The major problem related to the exploit of liposomes is their impaired colloidal stability, which can be overcome by coating them with hydrophilic polymers. The one proposed by Knudsen et al. (2012) was poly(ethylene glycol) (PEG). Liposomes loaded with calcipotriol were prepared by the thin film method, and in their bilayer membrane, different molar ratios of PEG_2000_-DSPE were included. The colloidal stability of the liposomes was received as a result of the inclusion of 0.5, 1, and 5 mol % PGE-DSPE in the membrane. PEGylation has been shown as a promising approach for stabilising calcipotriol-containing liposomes and improving skin penetration [61].

Earlier studies demonstrated that there is no remarkable improvement in the action of liposomal-incorporated vitamin D3 analogues compared to the free analogues related either to their proliferation or their IL1 alpha-releasing effects on keratinocytes. However, the authors suggested that the influence of liposomal-incorporated VD3 analogues in keeping small their adverse side effects has to be investigated at a more relevant model [60]. Results presented by Körbel et al. (2001) definitely suggest that using liposomal calcitriol and tacalcitol allows receiving a better anti-psoriatic effect compared to non-liposomal phospholipid gel and conventional formulations that are clinically used, i.e., ointments based on petrolatum. The authors suggested that, by using a liposomal formulation, the concentration of the analogue could be reduced compared to that of currently available commercial preparations without sacrificing any therapeutic effect, and thus lead to a reduction both in local side effects (skin irritation) and systemic side effects (hypercalcemia). The advantage of using liposomes is their physicochemical similarity to the lipid bilayer [64].

Another interesting approach for effective delivery is using solid lipid nanoparticles. They have good biocompatibility, the ability to protect some labile compounds against degradation, and the ability to modulate drug release. The research conducted by Sonawane et al. (2014) was focused on preparing betamethasone and calcipotriol co-loaded solid lipid nanoparticles for the treatment of psoriasis. Nanoparticles were prepared by hot melt high shear homogenization technique and incorporated in Carbol gel matrix. The average particle size was 188 nm, and entrapment efficiency of betamethasone and calcipotriol was very high for both drugs, 85%, and 97%, respectively. The versatile in-vitro (rat skin permeation and dermal distribution, anti-proliferative effect in human hyperproliferative keratinocyte cell lines) and in-vivo analysis (anti-psoriatic mouse tail studies, transepidermal water loss, and Draize patch irritation) proved the high potential use for skin application and higher anti-psoriatic efficiency compared to the commercial preparation (gel containing non-encapsulated betamethasone and calcipotriol). It was demonstrated for solid lipid nanoparticles that the drugs penetrated to epidermal and dermal skin layers, but were detected only in negligible amounts in the receptor compartment. Additionally, in in-vivo studies, nanoparticle-loaded gel significantly decreased the epidermal thickness and increased melanocyte count in comparison to commercial gel [65]. The work of Sonawane and co-workers is proof that drug delivery systems can provide safer and more effective anti-psoriatic therapy than conventional formulations.

On the contrary, in the study of Lalloz et al. (2018), it was not possible to penetrate VD into the epidermis through the intact pigskin with the use of poly(lactic acid) nanoparticles, regardless of the surface chemistry, although, the average particle size of nanoparticles was about 100 nm [66].

Finally, a different type of polymeric nanoparticles for local VD delivery was presented by Ramezanli et al. (2017). ABA triblock copolymers composed of hydrophilic A blocks and hydrophobic B blocks were used for the preparation of TyroSpheres. The polymers self-assemble in an aqueous environment to form a core-shell structure with a hydrophobic core in which VD3 is incorporated with hydrophilic blocks, making the shell and stabilizing the system. The average diameter was approximately 70 nm, and the encapsulation efficiency was high. VD3 was released from TyroSpheres in a sustained manner and was delivered across the stratum corneum separated from human cadaver skin. An ex-vivo skin distribution study showed that TyroSphere formulations delivered significantly higher amounts of active into the epidermis compared to VD dissolved in Transcutol^®^. Additionally, TyroSpheres protected the vitamin against photodegradation and hydrolysis [52].

## 4. Drug Delivery Systems for the Prevention and Treatment of Cancer

Increasing evidence supports the role of VD systems in cancer prevention and therapy. Many scientific articles have reported the role of VD systems in the onset, progression, prognosis, and treatment of cancer. The mechanisms of the genomic action of VD systems seems to be multidirectional.

**Figure 4:**
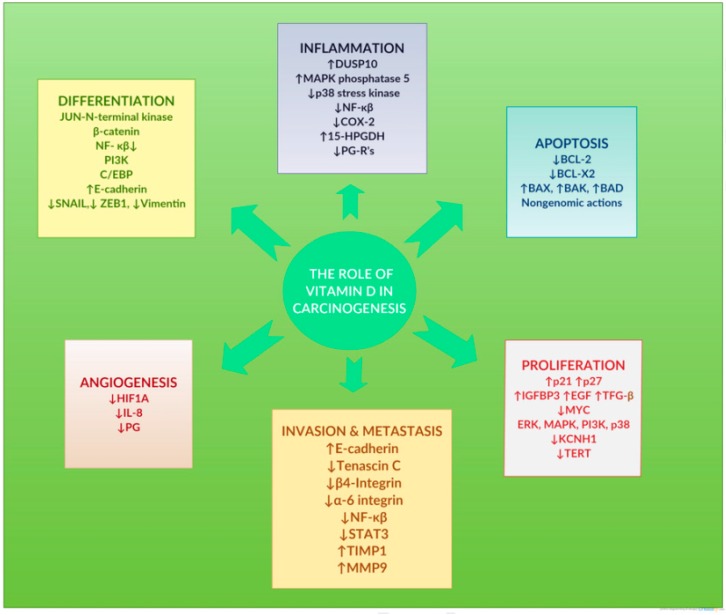
Preclinical basic-sciences studies show that the vitamin D system coordinates vital cellular processes such as cell differentiation, modulation of inflammation, apoptosis, cell proliferation, invasion, and metastatic processes, and angiogenesis. Reproduced with permission from [8], published by Elsevier, 2017.

Since the first hypothesis linking VD with possible anticancer activity in the early 1980s, a vast number of preclinical and clinical studies have been carried out, and various mechanisms of VD system actions in carcinogenesis have been reported in thousands of articles [5,7,8,9,67,68]. Non-specific, transversal anticancer actions of VD systems are presented in Figure 4. However, the possible actions of VD systems implicated in cancer development may be exerted upon specific histologic subtypes of cancer, including mainly breast, prostate, and colorectal cancer cells. Accumulating results from preclinical and some clinical studies strongly suggest that VD deficiency increases the risk of developing cancer and that avoiding deficiency and adding VD supplements might be an economical and safe way to reduce cancer incidence and improve cancer prognosis and outcome [5,8].

In the review of Pandolfi et al. (2017), some forms of cancer are mentioned that benefit from a VD supplementation during treatment. For example, it has occurred that women with triple-negative breast cancer have lower cathelicidin antimicrobial peptide (CAMP), which is an essential cytotoxic and proapoptotic peptide. However, if the cells are stimulated with calcitriol, the level of CAMP rises, leading to potential advantages for treatment. Another positive effect and a new therapeutic avenue in breast cancer treatment may also be observed as a result of VD inhibition of the cluster of microRNA-199a/microRNA-214, a well-known tumor promoter. Aside from that, research in Norway has shown that patients who have prostate cancer had better outcomes during summer when sunlight exposure is higher as well as VD serum levels. A similar situation was observed in connection with the morbidity of colorectal cancer. Despite using different case populations, risk factors, and various techniques of VD measurement, the studies confirmed the inverse correlation between VD intake and colorectal cancer risk [9].

### 4.1. Vitamin D as an Adjuvant in Cancer Therapy

An interesting suggestion might also be a combination of VD with other active substances. Xu et al. (2018) investigated the synergistic effect of astemizole and VD in hepatocellular carcinoma cells in vitro and in vivo. Both actives were used as non-encapsulated. It has been proven in vitro that astemizole enhances VD-induced apoptosis and decreases cell proliferation and migration. However, the decrease of tumor number, mass, and incidence in hepatocellular carcinoma cells was demonstrated in vivo. Astemizole increased VDR expression both in cells in vitro and in tumor tissues in vivo. They also clarified that this combination inhibits the tumor suppressor miR-125a-5p and is responsible for subsequent upregulation of the VD receptor. These outcomes highlight the crucial role of combined treatment of astemizole and VD for the cure of hepatocellular carcinoma cells [69]. Similarly, in other studies, VD was encapsulated into nanostructured lipid carriers and was used as an adjuvant to elevate the efficacy of doxorubicin on concurrent administration in breast cancer treatment. The prepared lipid nanoparticles revealed a mean particle size of 87 nm and were in the range for passive targeting to cells. In-vitro cytotoxicity results exhibited that VD-loaded nanoparticles were more effective than free VD in the induction of breast cancer cell death after 24-h incubation. Indeed, cotreatment of the cells with VD-loaded nanoparticles and doxorubicin caused a twofold increase in the percentage of apoptosis [70]. An innovative approach was presented by Maayah et al. (2018). They synthesized doxorubicin-VD by conjugating VD to doxorubicin to increase the delivery of doxorubicin into human osteosarcoma cancer cells and mitigate the chemoresistance associated with doxorubicin. The results were very promising, as the conjugate doxorubicin-VD suppressed the growth of cancer cells by inducing apoptosis while inhibiting cell survival and proliferative signaling pathways. They proposed that VD may serve as a novel drug delivery approach to potentiate the delivery of anticancer drugs into cancer cells [71].

### 4.2. Vitamin D and Its Metabolites as Anticancer Drugs

Particularly in the case of anticancer application, drug delivery systems can be useful due to the possibility of selective drug delivery to tumor tissue either by passive targeting with the enhanced permeability and retention effect or by active targeting using functionalization. In this way, nanoparticles, when internalized by target cells, increase intracellular drug delivery, sustain drug release, increase therapeutic activity, and lower side effects. It is especially important knowing that calcitriol has anti-tumoral activity in supraphysiological doses which are associated with a high risk of hypercalcemia. Beyond very high toxicity, calcitriol has a very short half-life in bloodstream; this is why its administration is particularly problematic [72,73]. In the case of cancer therapy, parenteral administration of nanoparticles like intravenous injection/infusion or direct injection to the tumor are the most probable way of administration since the oral route requires additional resistance of drug and drug carriers to such factors as gastric juice and enzymes. From a variety of drug delivery systems, liposomes have been considered so far as the most successful drug delivery system as liposome-based drugs have been clinically used in cancer therapy (e.g., Doxil^®^, DaunoXome^®^, Depocyt^®^, Mepact^®^, Myocet^®^).

It is essential to mention studies prepared by Almouazen et al. (2013) where they encapsulated VD, calcidiol, or calcitriol into poly(lactic acid) (PLA) nanoparticles. As expected, encapsulation into PLA nanoparticles significantly improved the stability of VD, calcidiol, and calcitriol compared to non-encapsulated forms. Free or nanoencapsulated drugs were dispersed in cell culture medium (Dulbecco’s Modified Eagle Medium with antibiotics) and incubated at 37 °C. After 24 h, more than 75% of the drugs remained in nanoparticles, whereas only about 25% of free VD and calcitriol remained in the medium, and free calcidiol was already totally degraded. In this study, loaded nanoparticles showed similar growth inhibition of human breast adenocarcinoma cells to non-encapsulated calcidiol and calcitriol [72]. This was not surprising, as calcidiol and calcitriol are lipophilic and readily cross the membranes to reach their nuclear receptors. A similar approach was presented by Ramalho et al. (2015). They prepared poly(lactic-*co*-glycolic-acid) (PLGA) nanoparticles as nanocarriers for calcitriol with a mean diameter of 186 nm. The in-vitro cytotoxic effects on three different human cell lines (two non-tumor pancreatic cell lines and one lung cancer cell line) after treatment with calcitriol-loaded nanoparticles were assessed relative to free calcitriol in terms of cell growth. Both free and encapsulated calcitriol exhibited a similar concentration-related decrease in cell growth and survival of the human non-tumor cells. However, calcitriol-loaded nanoparticles demonstrated an increased inhibitory effect on lung cancer cell line by inducing cell cycle arrest and morphological changes in the tumor cells [73].

A different approach was proposed by Vora et al. (2017), who created PLGA microspheres (28 µm) loaded with VD as an injectable controlled drug delivery system. The results showed that these microspheres could offer an interesting method for once-a-month delivery by the parenteral route (intramuscular injection), which was confirmed by in-vivo studies in rats [74]. However, such an approach could be an alternative for daily oral supplementation of this vitamin rather than a way to induce anticancer effects since such a system does not protect from hypercalcemia. On the other hand, Nguyen et al. (2007) reported a promising technique in liver cancer treatment by utilizing crosslinked microspheres (35 µm) prepared by polymerization as a carrier to control the release of calcitriol at the targeted site over a long period after direct injection of microspheres into the tumor. Local treatment through hepatic injection of calcitriol should prevent the development of hypercalcemia [75].

### 4.3. Active Targeting of Nanoparticles Loaded with Vitamin D to Cancer Cells

Very interesting studies were done by Liu et al. (2018). Calcitriol is also known as a promising agent for the treatment of non-small cell lung cancer with a developed resistance to inhibitors of EGFR (epidermal growth factor receptor) tyrosine kinase. However, calcitriol induces the expression of 24-hydroxylase, the enzyme that decreases calcitriol activity. That is why CTA091, a potent and selective 24-hydroxylase inhibitor, has been developed; however, CTA091 also suppresses renal 24-hydroxylase activity and so may promote hypercalcemia. To exploit the favorable effects of both calcitriol and CTA091 in tumor cells, tumor-targeted co-delivery of calcitriol and CTA091 was proposed by developing EGFR-targeted liposomes loaded with both drugs. Targeting of liposomes was achieved by conjugating human anti-EGFR monoclonal antibody to the liposomal surface. Liposomes were produced as uniformly distributed nanoparticles, with a mean diameter by volume of 157 nm and positive zeta potential. Compared with free calcitriol and CTA091, such targeted liposomes with co-loaded drugs have shown improved cellular uptake and effective inhibition of colony formation in lung cancer cells [76].

It should be mentioned that in some studies, conclusions suggest that VD is ineffective in cancer prevention/treatment and its role is overestimated. The research conducted by Maleklou et al. (2016) proved that targeted delivery of VD-loaded nanoparticles to C6 glioma cell lines reinforces the resistance to doxorubicin, epirubicin, and docetaxel and increases the cancerous characteristics in vitro [77]. It is also speculated that VD intake can be more significant in early carcinogenesis stages rather than advanced cancers [5]. Besides, many trials had some limitations, including being largely unable to observe a statistical relevant reduction in cancer occurrence or a lack of knowledge about the appropriate dose, the form of VD, and the length of time that it should be taken [10]. The large-scale and long-term human randomized controlled trials are not available so far, and there is no clear conclusion whether vitamin D can be beneficial during cancer treatment. Based on existing studies, it is crucial to avoid VD deficiency; VD might play a role in cancer risk reduction and treatment [5].

## 5. Drug Delivery Systems for Other Diseases or Routes of Administration

As mentioned earlier, VD was demonstrated to have multiple extra-skeletal effects on biological processes, such as immune regulation, neurogenesis, or genome stability, and the level of it can be associated with the therapy of other illnesses. Recent studies have focused on how VD can be exploited in the treatment of chronic, infectious, or autoimmune diseases. Some scientists also presume that we should consider VD as a hormone rather than a nutritional vitamin [78].

The immunomodulatory effects of VD through regulation and differentiation of lymphocytes, macrophages, and natural killer cells enhance opportunities to reinforce the treatment of arthritis. The research conducted by da Silveira et al. (2016) suggests that VD may inhibit the proinflammatory response. In the study, first arthritis was induced in rats, and then the groups were treated for 15 days with oral VD3 either in solution based on corn oil (120 IU/day) or VD3-loaded nanocapsules (15.84 IU/day). Lipid-core nanocapsules were made by interfacial deposition of preformed polymer technique using poly(ε-caprolactone) as a polymer making the shell. The versatile analysis was done before and after treatment for all the groups including chronic inflammation evaluation, histopathological observation, measurements of the activity of the inflammatory state enzymes in rat lymphocytes, and determination of many hematological and biochemical parameters in blood. As a result, better anti-inflammatory activity was demonstrated for the oral oily solution of VD3. However, it should be mention that the dose of encapsulated VD3 was approximately more than 7.6-fold lower than the dose of non-encapsulated vitamin. While an increase in serum levels of 25OHD was observed only in groups receiving non-encapsulating VD3, histological analyses showed that both formulations were able to reduce the inflammatory changes [79].

Asthma is another immunologic disease characterized by chronic airway inflammation that is a promising target for VD. In the research done by Wei-hong et al. (2014), nanoemulsions were prepared for the delivery of VD in ovalbumin-induced asthmatic mice, and VD bioavailability and anti-inflammatory activity were evaluated. In the study, nanoemulsions were prepared from the established composition of solid self-emulsifying drug delivery systems (sSEDDS) by spray-drying technique. VD in the form of either the non-encapsulated one or nanoemulsion was administered to mice with induced asthma at a high dose of 2000 IU/kg. It was evident that nanoemulsions significantly increased oral VD absorption. This increase in bioavailability resulted in enhanced pharmacological activity. The anti-inflammatory and antioxidant assay revealed that the therapeutic efficiency of VD significantly enhanced upon nanoemulsion formation [80].

An interesting approach was presented by Goff et al. (2012). The purpose of this study was to evaluate whether β-glucuronides of 1,25(OH)D (calcitriol) could deliver free calcitriol to the colon to treat colitis while decreasing the risk of hypercalcemia. Several studies have demonstrated a direct therapeutic effect of VD and calcitriol in mouse models of inflammatory bowel disease, since VDRs are widely expressed in the epithelial cells and the immune cells in the colon. The authors conjugated glucuronic acid to the calcitriol molecule to form water-soluble 1,25-dihydroxy vitamin D-25-β-glucuronide that is inactive, and little of that glycosidic form can be absorbed across the intestine. Cleavage of the β-glucuronide of the calcitriol to free calcitriol upon hydrolysis by β-glucuronidase produced by bacteria in the lower intestinal tract would: (i) Reduce systemic absorption of the 1,25(OH)D, (ii) decrease hypercalcemic effects, and (iii) allow targeted delivery of 1,25(OH)D to the ileum/colon cells affected by the disease. As a result, the authors confirmed that β-glucuronides of vitamin D compounds delivered 1,25(OH)D to the lower intestine and reduced hypercalcemia and symptoms of acute colitis in this model [81].

The influence of VD on immune and anti-inflammatory regulation raises the question of potential benefit against bacterial infections. Therefore, the study presented by Castoldi et al. (2017) was focused on the encapsulation of calcifediol in liposomes to enable aerosolization. Prepared 25(OH)D-loaded liposomes were nanosized and monodisperse, with a negative surface charge and a 25(OH)D entrapment efficiency of approximately 23%. Jet nebulization of liposomes was seen to yield an aerosol suitable for tracheobronchial deposition. Calcifediol in either liposomes or ethanolic solution was tested for the capacity to prevent pulmonary infection by *Pseudomonas aeruginosa*. Interestingly, encapsulation into liposomes resulted in a significant reduction in bacterial survival. Besides, the formulation can be regarded as a means to achieve calcifediol lung deposition and as safe for pulmonary administration in humans [82].

In recent years it has been found that low serum 25-hydroxyvitamin D3 concentration is connected with periodontal destruction in periodontitis and likewise with osteoporosis in diabetes. Both studies conducted by Li et al. (2013, 2014) endeavoured to prove that 25OHD-loaded PLA microspheres may ensure a potential therapy for patients with periodontitis. These microspheres were prepared using an emulsion-solvent evaporation method and were evaluated due to the biological effect on both in-vitro and in-vivo models. As a result, drug release during these experiments remained almost steady, which is promising for long-term therapy. The 25OHD-loaded PLA microspheres upregulated VD receptor expression and impaired periodontal inflammatory infiltrate and bone loss in diabetic rats with periodontitis [83,84].

Furthermore, a growing body of evidence has pointed out that calcitriol, an active form of VD, takes a role in maintaining brain functions through stimulation of neurotrophic factors’ expression, immune regulation, and prevention of neuronal damage. It is worth mentioning the approach made by Ślusarczyk et al. (2016), who prepared nanocapsules with calcitriol for neuroprotective targeted therapy to the brain and compared protective properties of calcitriol-loaded nanocapsules with those of non-encapsulated calcitriol in hippocampal organotypic cultures after treatment with lipopolysaccharide (LPS). The active substance was enclosed in a hydrophobic core coated with either PLL (poly(l-lysine hydrobromide)), PLL/PGA (poly(l-glutamic acid)), or PLL with pegylated PGA. The average size of nanocapsules ranged from 80 to 100 nm. The results demonstrated that carriers with PLL/PGA and PLL-PGA-PEG were recognized as non-toxic and as having a protective ability. Both forms of nanoparticles also exhibited a higher neuroprotective action than free calcitriol connected with the suppression of LPS-induced nitric oxide (NO) release. Since calcitriol can modulate the brain NO signalling, it may be used as a promising solution in neuroinflammation and neurodegenerative processes [85].

## 6. Conclusions

Due to its many beneficial effects, the interest in VD and its derivatives has increased, and they have become attractive as active substances for incorporation into DDS. In this review, we summarized and discussed the main directions of development of drug delivery technologies that have been reported in recent years for VD or VD analogues. Overall, although the number of works describing new technologies appears large at first glance, and they cover a wide range of strategies, they are all at an early stage of research. In this work, many types of DDS have been presented depending on the disease or route of administration. Oral administration is the most convenient route, and is cost-effective, to increasing 25(OH)D levels, especially since large proportions of the population have low VD levels. The implementation of DDS for food enrichment or VD therapy with dietary supplements seems to be justified and feasible in the near future. However, most interesting is the potential use of VD/VD analogues in anticancer therapy. The combination of both strategies, i.e., synthesis of low calcemic VD analogues and targeted delivery, seems to be most desirable. However, until now, no sophisticated technology with functionalization of DDS was introduced for VD or VD derivatives. Nevertheless, the delivery of VD-based drug payloads via tumor-targeted carriers seems to be a future direction.

## Figures and Tables

**Table 1:** Examples of various types of delivery systems reported for vitamin D encapsulation for potential use in food and beverage fortification (VD2—vitamin D2, VD3—vitamin D3, MCT—medium chain triglycerides).

Delivery System	Active	Main Components	Technique/Method	Reference
**O/W emulsions and microemulsions**	VD2 VD3	Oil phase: Soybean oil, olive oil, or MCT; water phase containing Tween 20 or sodium cholate	Microchannel emulsification	[19]
	VD2 VD3	Oil phase: Soybean oil, olive oil, or MCT; water phase containing Tween 20	Rotor-stator and high-pressure homogenization	[36]
	VD3	Oil phase: MCT; water phase containing Tween 20, 60, or 80	High-speed blender and high-pressure homogenization	[30]
	VD2	Oil phase: Soybean oil; water phase containing modified lecithin, sodium caseinate, or decaglycerol monooleate	Rotor-stator and high-pressure homogenization	[37]
**O/W nanoemulsions**	VD3	Oil phase: MCT, corn oil, fish oil, mineral oil, or orange oil; water phase containing a natural surfactant	High-speed blender and high-pressure homogenization	[34]
	VD3	Oil phase: Fish oil; water phase containing Tween 20	Ultrasonication	[38]
**Biopolymer-based nanoparticles**	VD3	Zein nanoparticles coated with carboxymethyl chitosan	Phase separation method and coating by cross-linking with calcium	[32]
	VD2	Beta-lactoglobulin–sodium alginate complex	Electrostatic interactions	[39]
	VD3	High amylose corn starch	Ultrasonication	[40]
	VD3	Carboxymethyl chitosan–soy protein complex	Ionic gelation method	[41]
**Lipid-based nanoparticles**	VD2	Solid lipid nanoparticles (glyceryl tripalmitate) stabilized by Tween 20	Hot homogenization technique using a high-pressure homogenizer	[42]
	VD3	Nanostructured lipid carriers (solid lipids: Precirol or Compritol, liquid lipid: Miglyol or octyloctanoat, surfactants: Tween 80 or 20 or Poloxamer 407)	Hot homogenization method	[43]
	VD3	Nanostructured lipid carriers (glycerol monostearate as solid lipid, oleic acid as liquid lipid, and Tween 80)	Hot high-pressure homogenization	[44]
**Micelles**	VD3	Amphiphilic chitosan derivative of *N*,*N*-dimethylhexadecyl carboxymethyl chitosan	Synthesis	[45]
**Liposomes**	VD3	Soybean phosphatidylcholine, cholesterol	Thin film hydration-sonication technique	[46]
**Microparticles**	VD2	Medium molecular weight sodium alginate	Ultrasonic atomization and microwave stabilization	[47]

**Table 2:** Examples of various types of delivery systems reported for encapsulation of vitamin D or its analogues for potential topical delivery (VD3—vitamin D3).

Delivery System	Active	Main Components	Technique/Method	Reference
**Liposomes**	Calcipotriol	Dipalmitoylphosphatidyl-choline (DPPC) and dilauroylphosphatidylcholine (DLPC)	Thin film method and extrusion	[63]
	Calcipotriol	Distearoylphosphatidylcholine (DSPC), poly(ethylene glycol)-distearoylphosphoethanolamine (PEG_2000_-DSPE), sodium cholate	Thin film method and extrusion	[61]
	Calcitriol and tacalcitol	Phosphatidylcholine, phosphatidic acid, phospha-tidylethanolamine	Made from concentrate (commercial kit)	[64]
**Solid lipid nanoparticles**	Bethamethasone and calcipotriol	Precirol^®^ ATO 5	Hot melt high shear homogenization technique and incorporation in Carbol gel matrix	[65]
**Polymeric nanoparticles**	VD3	Poly(lactic acid) nanoparticles with non-ionic poly(ethylene glycol) or zwitterionic poly(2-methacryloyloxyethyl phosphorylcholine) (PMPC) coating	Flash nanoprecipitation	[66]
	VD3	ABA triblock copolymers composed of hydrophilic A blocks and hydrophobic B blocks that form TyroSpheres^®^	TyroSpheres^®^ preparation	[52]
